# Electrolytic reduction of liquid metal oxides and its application to reconfigurable structured devices

**DOI:** 10.1038/srep08637

**Published:** 2015-03-02

**Authors:** Jinqi Wang, Kanagasundar Appusamy, Sivaraman Guruswamy, Ajay Nahata

**Affiliations:** 1 Department of Electrical and Computer Engineering, University of Utah, Salt Lake City, UT. 84112, USA; 2 Department of Metallurgical Engineering, University of Utah, Salt Lake City, UT. 84112, USA

## Abstract

Structured metallic patterns are routinely used for a wide variety of applications, ranging from electronic circuits to plasmonics and metamaterials. Numerous techniques have been developed for the fabrication of these devices, in which the metal patterns are typically formed using conventional metals. While this approach has proven very successful, it does generally limit the ability to reconfigure the geometry of the overall device. Here, we demonstrate the ability to create artificially structured metallic devices using liquid metals, in which the configuration can be altered via the electrolysis of saline solutions or deionized water. We accomplish this using an elastomeric mold with two different sets of embedded microfluidic channels that are patterned and injected with EGaIn and water, respectively. The electrochemical reaction is then used to etch the thin oxide layer that forms on eutectic gallium indium (EGaIn) in a controlled reproducible manner. Once the oxide layer is dissolved locally, the underlying liquid metal retracts away from the original position to a position where a new stable oxide layer can reform, which is equivalent to erasing a section of the liquid metal. To allow for full reconfigurability, the entire device can be reset by refilling all of the microchannels with EGaIn.

The ability to modify the configuration of a given device geometry is of great importance in a broad range of applications. As an example, in the field of metamaterials, it is the specific geometry that gives rise to the response^1^,^2^. In order to have in-situ flexibility in determining the device properties, it would be advantageous to be able to make changes to the geometry in a controlled, reversible manner via the application of a simple external stimulus. Typically, devices that incorporate a structured metallic pattern are fabricated by depositing and patterning thin layers of conventional metal films, such as gold, silver or aluminum. However, such an approach does not lend itself easily to enabling large-scale changes in the structure; in the case of metamaterials, structures that incorporate semiconductors^3^ or phase-change media^4^,^5^ have been shown to allow for small-scale changes in the geometry when exposed to an external stimulus.

One approach that is amenable to allowing for large-scale changes in the structure geometry involves the use of liquid metals. The most commonly used member of this family of materials, eutectic gallium indium (EGaIn), is composed of 78.6% Ga and 21.4% In by weight and has a melting point of ~15.5°C, making it liquid at room temperature. EGaIn forms a thin passivating oxide layer that enables the metal to form in non-spherical shapes^6^ and is non-toxic^7^. These two properties make the material particularly useful for a variety of stretchable devices, including antennas^8^, plasmonic devices^9^, fibers^10^, solar cells^11^ and 2D and 3D self-healing wires^12^. In the absence of this oxide layer, EGaIn behaves like mercury and contracts into a spherical shape, since both materials exhibit high surface tension^7^. In fact, we have recently shown that when metamaterials fabricated using EGaIn inside a polydimethylsiloxane (PDMS) microfluidic structure are exposed to an acid environment, the oxide layer is dissolved and the resulting bare liquid metal retracts away from the exposed area, effectively erasing the affected area^13^. In that case, we used HCl that was brought into contact with the PDMS surface and the embedded liquid metal oxide was etched away because of the porous nature of the elastomeric mold. While the approach was successful in erasing components of the liquid metal geometry, it suffered from several limitations: (i) the size of the HCl drop on the PDMS surface limited the minimum dimensions of the erased area (ii) the HCl exposure time varied depending upon the thickness and porosity of the PDMS layer (iii) the erased area was dependent on the thickness of the PDMS layer and (iv) repeated exposure to HCl degraded the elastomer, thereby limiting the number of erase/refill cycles that could be performed.

In this submission, we demonstrate that an electrolytic process can be used to change the geometry of a liquid metal-based structured device in a more localized and controlled manner. To accomplish this, we fabricate a device that incorporates lithographically defined electrodes and two different sets of microfluidic channels within an elastomeric PDMS mold fabricated using conventional soft lithography techniques. We fill one set of channels with EGaIn, forming an array of closed rings. The second set of channels is filled with water, either deionized or as a dilute saline solution, and is in contact with both the liquid metal and the electrodes. By applying a voltage between different electrodes, we create a highly localized reducing environment around the EGaIn channel region closest to the positive electrode, which dissolves the oxide layer in a controllable, reproducible manner. This EGaIn region effectively serves as the cathode. When the external voltage is removed, the electrolytic process ends abruptly. This approach can be used to erase only portions of individual close rings or it can be performed in parallel to simultaneously etch multiple rings, each at a different rate and in a selective manner (i.e. each ring can be individually addressed). After etching, the entire device can be refilled with EGaIn, effectively allowing for a complete reset of the device. Since the localized electrolytic cell volume is small and the voltage applied for only short periods of time, the process can be cycled without any noticeable degradation to the PDMS structure. The approach is sufficiently general that it can be applied to a wide variety of other geometries and applications, including reconfigurable antennas and electrical circuits.

## Results and Discussion

**Figure 1:**
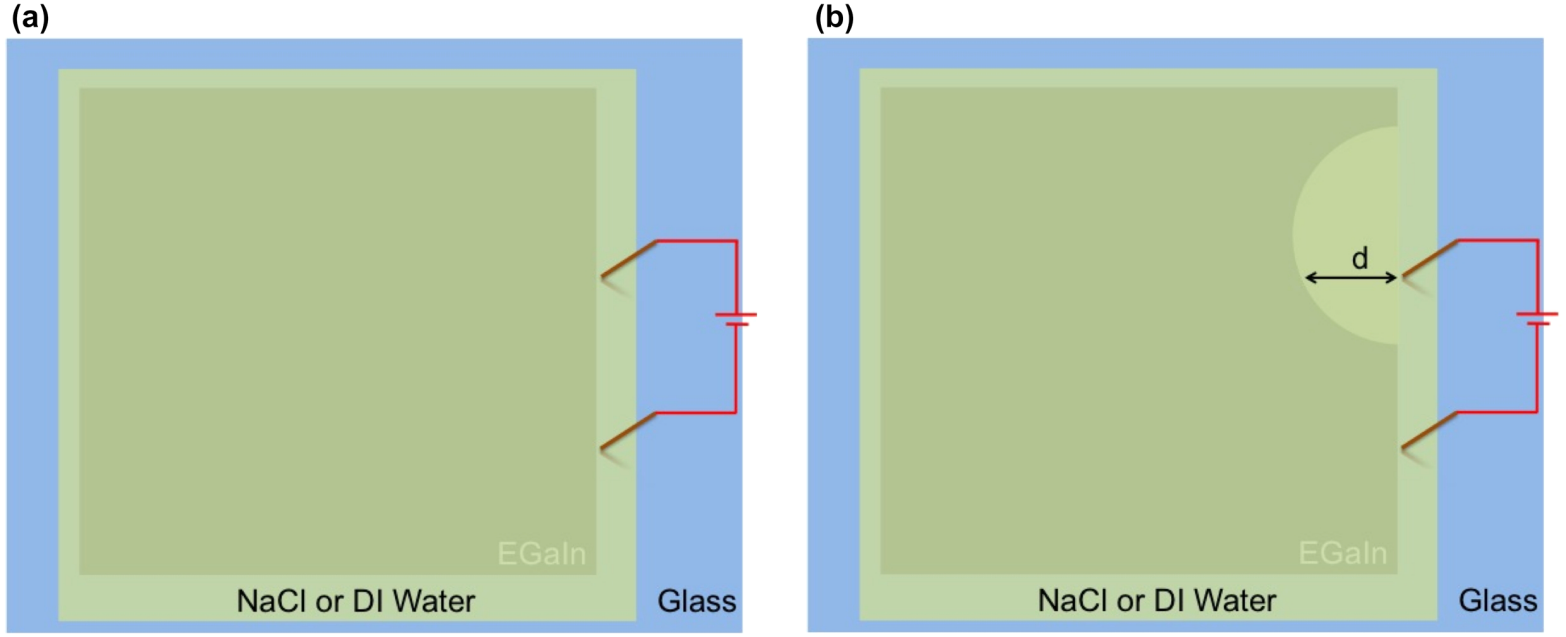
Schematic diagram of the geometry used for measuring the electrolytic process. (a) Geometry before the application of an external voltage (b) Geometry of the etched liquid metal after application of an external voltage showing the distance d used to measure the erasure rate. The upper electrode serves as the anode.

In order to explain how electrolysis can be used to create reconfigurable structured devices, we first discuss how the electrochemical process can be used to dissolve the oxide layer that forms on EGaIn. As noted above, when the oxide is etched, the exposed EGaIn quickly retracts to a position where a stable new oxide layer can form (i.e. a position where the extent of the reducing environment created is insufficient to remove/etch the oxide layer), corresponding effectively to erasure of the metal pattern in the exposed area^13^. In Figure 1, we show a schematic diagram of the approach used to measure the erasure rate of the liquid metal. A drop of EGaIn was placed on a clean glass slide and spread using the sharp edge of a second glass slide until a thin shiny film was formed. While the approach yielded a liquid metal film that had non-uniform thickness, the etching process only applied to the oxide layer that was expected to have a uniform thickness^14^. We then covered the metal film and the rest of the glass slide with a layer of water that contained different concentrations of NaCl. Two gold-coated copper electrodes, separated by 2 cm, were then placed in contact with the water layer and close to one edge of the metal film. With the application of an external voltage, EGaIn was only erased about the anode (i.e. the gold-coated copper electrode to which the positive voltage was applied), with no change in the liquid metal around the other gold-coated copper electrode. Furthermore, the erasure configuration was found to not be symmetric about the anode (i.e. not a semi-circle centered at the anode), but rather somewhat closer to a semi-oval with a center that was offset from the positive electrode, as shown schematically in Figure 1.

**Figure 2:**
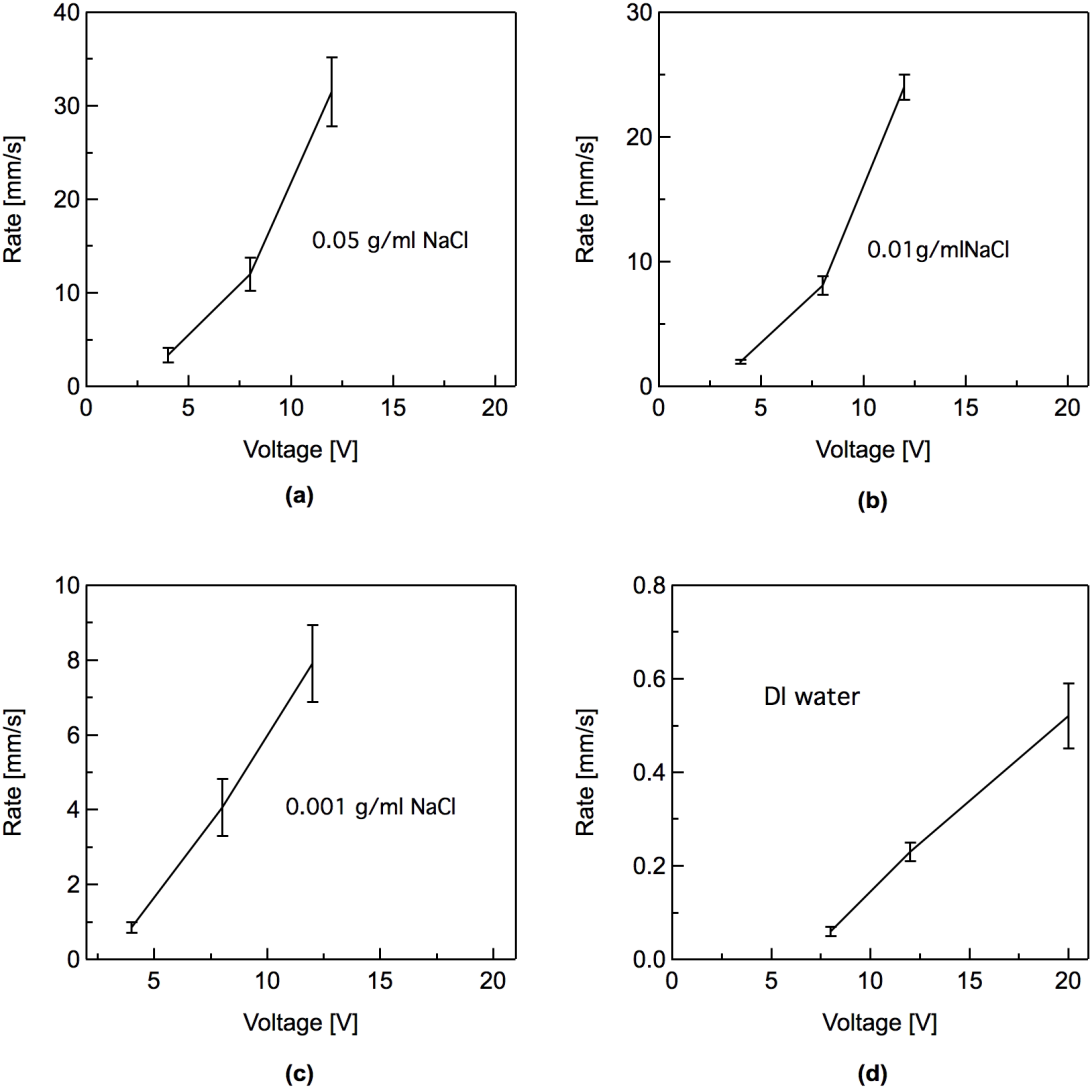
Erasure rates of EGaIn film using water with differing concentrations of NaCl. (a) 0.05 g/ml NaCl, (b) 0.01 g/ml NaCl, (b), 0.001 g/ml NaCl, and (d) deionized water.

To quantify the erasure process, we took videos of the erasure process and defined the erasure rate as the distance, d (as shown in Figure 1), erased per unit time after application of an external voltage. For each solution and each voltage setting, we performed multiple measurements with fresh samples to ensure reproducibility in the data. In Figures 2a–2c, we show the erasure rate for saline solutions containing three different NaCl concentrations for multiple voltages. In general, a larger applied voltage yielded a faster erasure speed. This is reasonable, since a higher voltage corresponds to higher current, which is expected to accelerate the electrolytic process. For very low NaCl concentrations, ~0.001 g/ml, the erasure rate appeared to vary approximately linearly with the applied voltage. However, for higher NaCl concentrations, the rate became increasingly nonlinear. Once the voltage was removed, the erasure process stopped almost immediately.

Although saline solutions can yield very fast erasure rates, use of the electrolyte also creates several significant challenges. First, as water in the channels evaporates, the salt concentration increases. Thus, the erasure rate changes over time. Furthermore, the channels need to be flushed regularly to prevent crystallization of NaCl on the channel walls as the water evaporates, which severely degrades the device performance. To avoid these issues, we tested the erasure process using only deionized (DI) water, with a resistivity of ~18 MΩ·cm and the same experimental configuration shown in Figure 1. The erasure rate, shown in Figure 2d, was significantly lower than for any concentration of saline solution tested.

**Figure 3:**
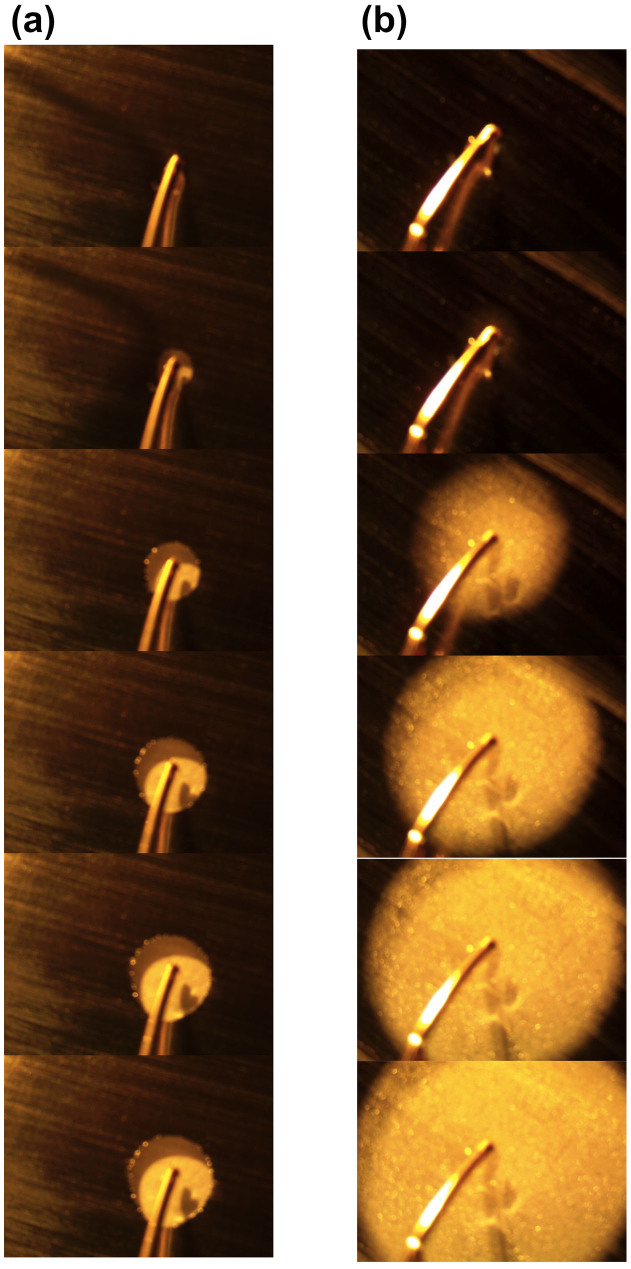
Snapshots from videos taken of the EGaIn erasure process using DI water and a saline solution. (a) Snapshots using DI water taken from Supplementary Video 1. The time interval between each snapshot is ~3 seconds. (b) Snapshots using a 0.001 g/ml NaCl solution taken from Supplementary Video 2. The time interval between each snapshot is ~350 milliseconds. In both cases, the anode was placed in the middle of the liquid metal film and the cathode was placed at the periphery of the film in the aqueous solution.

The test configuration in Figure 1, where the electrodes are close to one edge of the EGaIn film, is very close to the one that we adopt for the structured devices described below. To visually compare the erasure rates using DI water and saline solutions, we show time-stepped snapshots captured from videos taken of the erasure process in Figures 3a and 3b (supplementary video 1 corresponds to DI water and supplementary video 2 corresponds to a saline solution with a NaCl concentration of 0.001 g/ml). In both cases, the anode electrode was placed in the middle of the EGaIn film. The time interval between consecutive snapshots is ~3 seconds in Figure 3a and ~350 milliseconds in Figure 3b, confirming the dramatic differences in oxide etch rates.

To describe the etching process, we first discuss the properties of the oxide film. Cademartiri and co-workers used x-ray photoelectron spectroscopy (XPS) and its variants to study the composition of this heterogeneous film^14^. Using XPS, they found signatures of three different types of oxide within the thin surface covering of the liquid metal: Ga_2_O_3_, Ga_2_O and In_2_O_3_, with Ga_2_O_3_ forming the dominant component. Using angle resolved XPS, they found that on average, the Ga_2_O_3_ formed the outer surface of the oxide. In the present work, in the absence of an applied potential, we would expect similar oxide formation when EGaIn comes into contact with either oxygen or water (pH ~ 7). We note that while the addition of NaCl changes the conductivity of the water, it does not alter the pH, so the following results and discussion apply to all of the experimental implementations discussed here.

**Figure 4:**
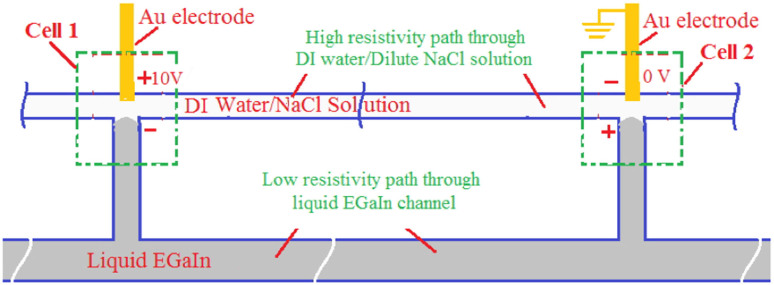
Schematic diagram of a representative electrochemical cell configuration for selective oxide removal. This specific geometry shown here has been chosen since it is relevant to the later discussion on a structured device. See the text for details.

When EGaIn or any other metal is immersed in a solution (DI water or a saline solution), an electrified interface is formed due to the electron transfer across the interface, associated with the corresponding electrochemical reaction, and the establishment of a electrical double layer^15^. The electron transfer accompanying the reaction at the electrode/solution interface leads to the development of a potential difference between the electrode and solution across the double layer. An equilibrium is established when the net electron transfer is zero or the oxidation and reduction rate at this interface is equal. The potential of the electrode under this equilibrium condition is measured against a non-polarizable reference electrode (a standard hydrogen electrode whose potential is taken as zero) and this potential is referred to as the equilibrium potential of EGaIn corresponding to this solution^15^,^16^. When the potential of the electrode deviates from this equilibrium value (due to an externally applied voltage or contact with another metal/material immersed in the same solution), the deviation is referred to as an overvoltage. An oxidation reaction occurs when the overvoltage is positive, in which case the electrode is referred to as the anode. Conversely, a reduction reaction occurs when this potential deviation is negative, in which case the electrode is referred to as a cathode. An electrochemical cell contains two half cells, one containing the anode and the other containing the cathode. The total cell voltage consists of the potential drop from the anode to the solution across the electrified double layer, the potential drop in the electrolyte between the electrodes, and the potential drop from the solution to the cathode across the electrified double layer. With the device configuration described here, the resistivity of the DI water and any of the NaCl solutions are high relative to EGaIn. Thus, the system can be modeled as consisting of two complementary electrochemical cells as shown in Figure 4. In cell 1, the Au electrode at positive voltage serves as the anode and the EGaIn in the adjacent channel serves as the cathode. Both DI water and NaCl solutions have a pH value of about 7. Thus, at this pH level, the equilibrium potential E_0_ for Ga_2_O_3_ reduction reaction is = −0.452 − 0.0591 × pH = −0.865 V. Similarly, the equilibrium potential E_0_ for In_2_O_3_ reduction reaction is = −0.181 − 0.0591 × pH = −0.594 V, again at a pH of 7. As the applied voltage is increased, the EGaIn electrode overvoltages corresponding to Ga_2_O_3_ and In_2_O_3_ reduction reactions in Cell 1 becomes increasingly negative and the reduction rate increases. Considering the presence of both Ga_2_O_3_ and In_2_O_3_ phases in the oxide layer on EGaIn, the oxide film reduction reactions at the EGaIn electrode (cathode) can be written as^17^
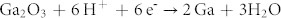

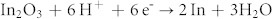
When the electrode potential is lower than the equilibrium potential, a reduction of the oxide film will occur. In addition, as the NaCl concentration is increased, there is a corresponding increase in the ionic strength and ionic conductivity. Thus, as the concentration increases, the potential drop in the solution across the two electrodes decreases further and is accompanied by corresponding increases in the anodic and cathodic overvoltages. The cell current values will increase exponentially with overvoltage and there will be corresponding increases in the oxide reduction rates. It is important to note that the negative overvoltages required for the reduction to metallic Ga and In are much smaller than the cell voltages applied. The need for such large applied voltages was necessitated by the large potential drops in the solution due to the relatively high resisivities of DI water and the different dilute NaCl solutions. These various effects are reflected in the observed erasure rates of the Ga oxide shown in Figure 2 (a–d).

**Figure 5:**
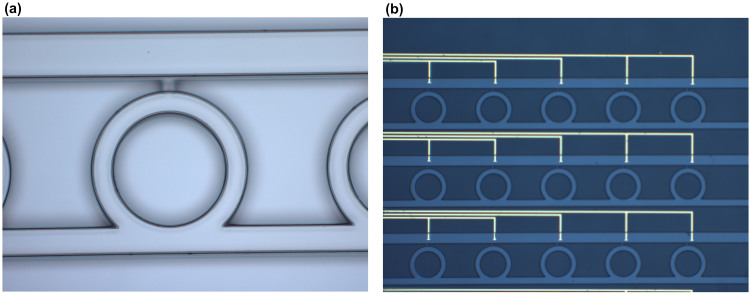
Images of the master and fabricated device. (a) Microscope image of a portion of SU-8 mold. The inner radius of the circular ring is 100 μm. The outer radius of the circular ring is 130 μm. The length of the vertical short channel is 20 μm. The width of upper horizontal channel is 72 μm. The width of the lower straight channel is 44 μm. The vertical periodicity in the unit cells is 450 μm, while the horizontal periodicity is 530 μm. (b) Microscope image of a portion of final device before injecting any liquids. The yellow lines are the Au electrodes.

The highly controllable erasure of EGaIn using electrolysis of water makes this an attractive approach for creating reconfigurable artificially structured devices. While almost any geometry can be used to demonstrate the utility of this approach, we have decided to create two separate arrays of closed rings embedded within a PDMS mold. The curved nature of the rings show clearly that the approach is not limited only to straight channels and can therefore be applied to a broad range of potential applications. The basic fabrication process relied on creating a master with the designed microfluidic channels using SU-8 photoresist, which was then transferred to PDMS using standard soft lithography techniques^18^. A microscopic image of one unit cell of the SU-8 structure is shown in Figure 5a. The lower horizontal channel and the ring structure were designed for the liquid metal. The upper horizontal channel and the short vertical channel above each ring were designed for DI water. With the exception of this short vertical channel above each ring, which had a height of 10 μm, all of the other channels were 32 μm in height. The 10 μm high short vertical channels were designed to have a smaller height because the pressure required to inject EGaIn into a microchannel varies inversely with its dimensions^7^,^19^. By having a smaller channel, the liquid metal was constrained to only the lower horizontal channel and the rings. On the other hand, since the surface tension of water is ~0.07 N/m, which is a much smaller value than for EGaIn, water easily filled the upper horizontal channel and the short vertical channels. The microscopic image of a portion of the device before injecting EGaIn and solution is shown in Figure 5b.

**Figure 6:**
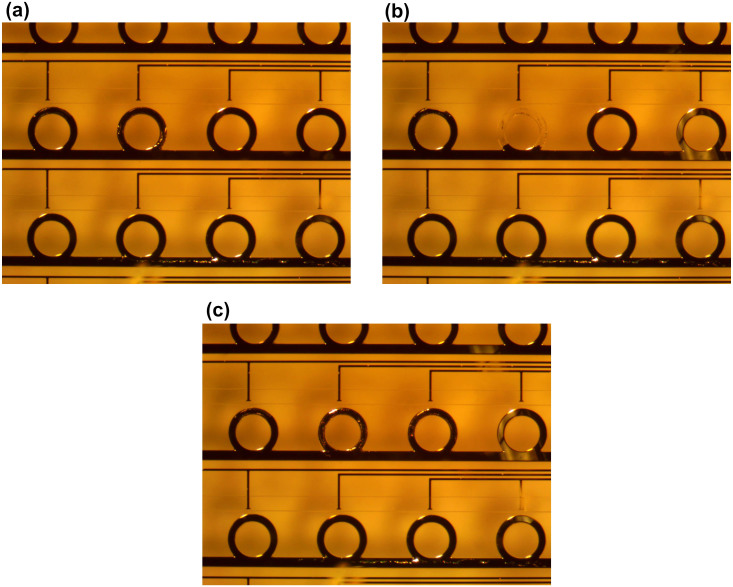
Snapshots of the erasure process within the CRR array. (a) A snapshot taken from Supplemental Video 3 before re-erasure of an EGaIn ring (after cycling between erasure and refilling several times). (b) Snapshot taken from Supplemental Video 3 after erasure of the middle EGaIn ring. A potential voltage of +10 V was applied to the Au electrode immediately above the CRR. The ground voltage was applied to an electrode well outside of the viewing window. (c) The snapshot after refill EGaIn back to microchannels. Please see the supplementary video to see the entire process.

To demonstrate the erasure process, we first injected EGaIn to fill all of the rings and lower horizontal channels. Then we injected DI water into the top horizontal channels, as well as the upper short vertical channels. In Figures 6a and 6b, we show snapshots of the erasure process before and after application of an external voltage (the complete erasure-refilling cycle is shown in supplementary video 3). To demonstrate this, we applied 10 V to two separated electrodes in the center row. A single protruding electrode (second from the left) within the observation window acted as the anode, while the cathode was outside of this window. The ring adjacent to the anode can be erased quickly. We note that because of the electrode geometry, electrochemical reactions are possible, though strongly suppressed, in adjacent unit cells. Further optimization of the water microchannel and gold electrode geometries are expected to mitigate this issue. Once the applied voltage is removed, the erasure process stops quickly, providing excellent control over the fraction of each ring that is erased. Since the electrolytic reduction of the oxide is highly localized and operates for only short periods of time, we found that there was no observable damage to the PDMS, even with repeated cycling. After selective erasure, EGaIn can be refilled into the microchannels to recover the original structure, as shown in Figure 6c, to achieve full reconfigurability.

**Figure 7:**
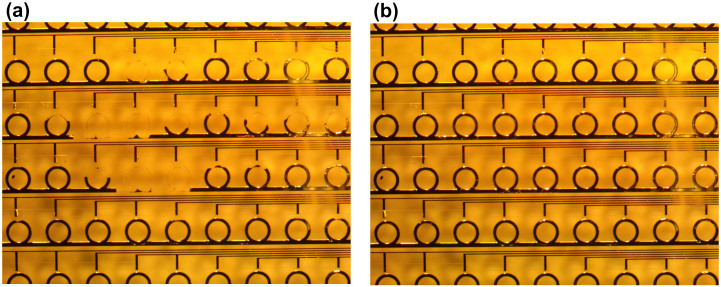
Snapshots of a video recording erasure of an array with random access. By controlling which electrodes have an applied voltage, the magnitude of that voltage, and the application time, we can selectively erase one ring or a group of rings each simultaneously, with the possibility of each ring being etched to a different extent. (a) Starting with a completely filled array, a voltage of +10 V was applied to the gold electrodes adjacent to the different rings for different amounts of time. If we consider the third complete row of rings, the voltage was applied to the third ring from the left for only half the time that it was applied to the fourth and fifth rings (corresponding to a half etched ring and two fully etched rings, respectively). Etching of the other rings can be explained in a similar manner. (b) The device after refilling EGaIn into microchannels to recover the original array. Please see the supplementary video to see the entire process.

Finally, we note that since the EGaIn rings can be electrochemically erased via the application of a localized external bias, the erasure process can be performed in either serial or parallel, to a single ring or to a group of rings in a random access manner. In Figure 7, we show the result of a multi-step process in which multiple rings in a larger array of closed rings are erased. In each step, we selectively erased one or several rings, although this could all be done in a single step. The fraction of each ring that is erased is determined by the both the applied voltage and the application time in each step. In contrast to earlier work, where large-scale structural changes lead dramatic changes in the optical response, each individual erasure step here would correspond to small geometry changes with correspondingly small changes in the response. The specific geometry change induced here is designed to show the extent of the changes possible and not to enable a specific response. In the context of electromagnetic measurements, the transmission properties of similar geometries using liquid metals have been shown in earlier work^13^,^19^. Therefore, we have not shown the spectral properties of these structures here. However, we expect them to be similar in nature.

## Conclusion

In summary, we have demonstrated that an aqueous electrochemical process involving the use of DI water with or without the addition of NaCl, can be used as an effective means of erasing liquid metals via etching of the oxide layer that stabilizes the shape of the material. To demonstrate this, we initially measured the erasure rate using water with varying concentrations of NaCl. We found that the erasure rate could be effectively controlled by both the applied voltage and the NaCl concentration. In general, higher voltages or higher concentrations of NaCl contributed to a faster erasure rate. Using this information, we fabricated a microfluidic device with embedded microchannels in the form of a closed ring array, as well as an adjacent geometry for various aqueous solutions. Using embedded gold electrodes, we demonstrated the ability to selectively erase one or a group of unit cells in a random access manner. This approach provides the capability to create large-scale geometry changes within a device that can be fully reconfigured. Although we used one specific device geometry, the approach can be extended to other geometries and other domains, such as reconfigurable antennas and circuits. Within the field of plasmonics and metamaterials, we expect this to create greater flexibility in designing a broader range of devices.

## Methods

### Fabrication of SU-8 template

We coated a silicon wafer coated with SU-8 3010 photoresist and spun it at 5000 rpm for 30 seconds. This film was exposed to UV light using a blank mask to form a flat layer, which was used to improve the adhesion between the silicon wafer and the upper SU-8 patterned layer. A second layer of SU-8 3010 photoresist was then spun cast at 3000 rpm. UV lithography using a mask with the structured pattern yielded a thin 10 μm photoresist layer with the designed pattern. A third layer of SU-8 3025 photoresist was spun at 4000 rpm above the second layer of SU-8 3010 photoresist. After baking, it was aligned and exposed to UV light using a mask with the CRR and two straight-line patterns. The third layer had a thickness of 22 μm. Both photoresist layers were then simultaneously developed to obtain the final two-layer pattern.

### Soft lithography

A PDMS pre-polymer was mixed with a curing agent using a volume ratio of 7:1, degassed, poured onto the SU-8 template, and cured for 2 hours at 60°C. After curing, the inverse PDMS replicas were peeled off and reversibly bonded with a glass substrate having patterned electrodes through van der Waals forces, yielding the desired microfluidic channel-based device.

### Embedded Electrodes for Electrolysis

We deposited a 10 nm layer of Cr and a 180 nm layer of Au on a clean glass slide. Using standard wet etch techniques, we patterned the metal to define electrodes. The patterned PDMS mold was aligned with Au electrodes and reversibly bonded with the glass substrate to form the final device.

## Author Contributions

A.N. and J.W. conceived of the idea and planned the project. J.W., K.A. and S.G. designed and carried out all of the electrochemical measurements. J.W. designed and fabricated the devices. All authors contributed to the scientific discussion and wrote the manuscript.

## Supplementary Material

Supplementary Information Supplementary Information

Supplementary Information Video 1

Supplementary Information Video 2

Supplementary Information Video 3

Supplementary Information Video 4
