# Supplementary material for: Electrolytic reduction of liquid metal oxides and its application to reconfigurable structured devices
Source: Sci Rep. 2015 Mar 2;5:8637. doi: 10.1038/srep08637 (PMC4345337; doi:10.1038/srep08637)
Supplement: Supplementary Information [file srep08637-s1.doc]

**Supplementary Information**

**Electrolytic reduction of liquid metal oxides and its application to reconfigurable structured devices**

**Jinqi Wang^1^, Kanagasundar Appusamy^2^, Sivaraman Guruswamy^2^, and Ajay Nahata^1,*^**

^1^ Department of Electrical and Computer Engineering, University of Utah, Salt Lake City, UT, 84112, USA.

^2^ Department of Metallurgical Engineering, University of Utah, Salt Lake City, UT, 84112, USA

^*^ Correspondence and requests for materials should be sent to A.N. (E-mail: [nahata@ece.utah.edu](mailto:nahata@ece.utah.edu))

**
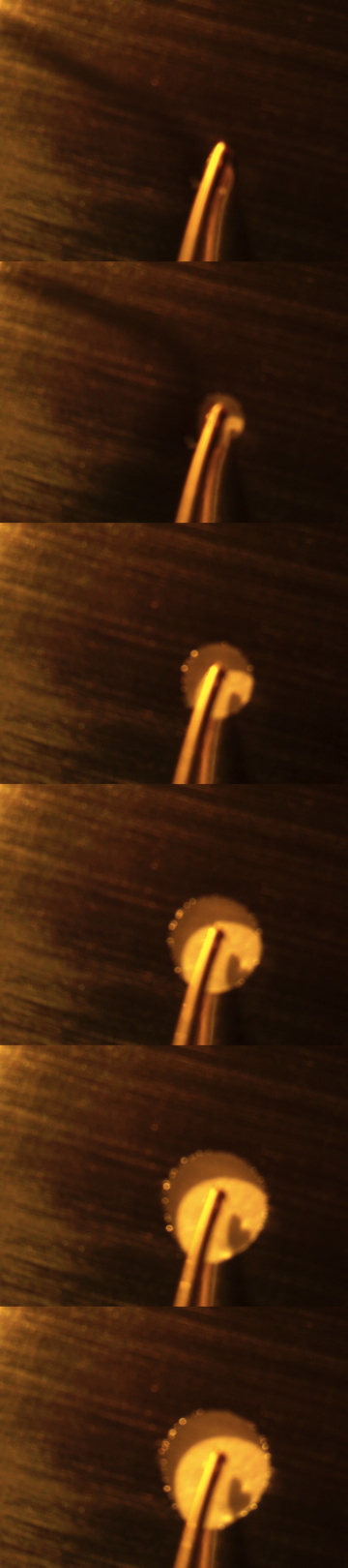
**

**Supplementary Video 1**. EGaIn erasure process using deionized water. The anode with an applied bias of +10 V was placed in the middle of the liquid metal film and the cathode was placed at the periphery of the film in the aqueous solution.


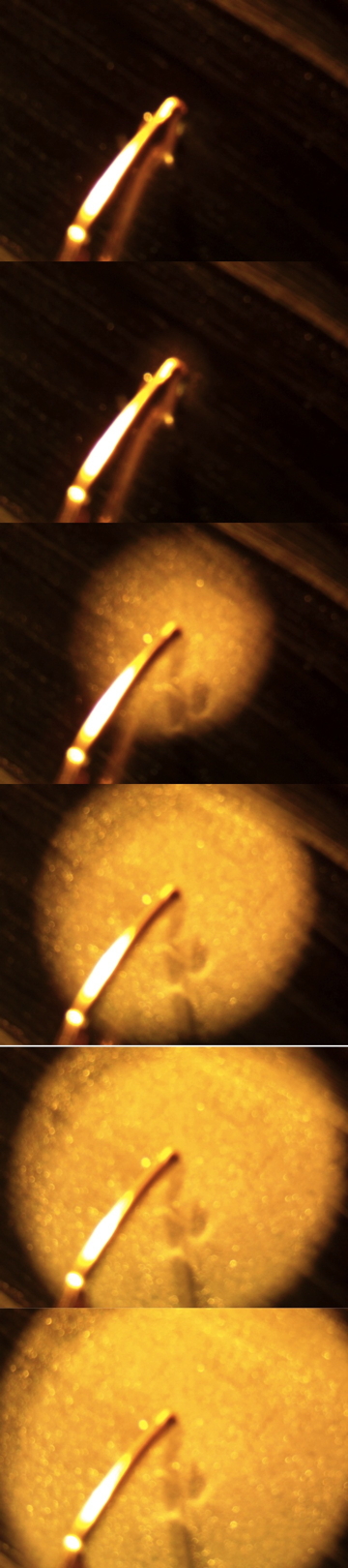


**Supplementary Video 2**. EGaIn erasure process using a saline solution (NaCl concentration of 0.001 g/ml). The anode with an applied bias of +10 V was placed in the middle of the liquid metal film and the cathode was placed at the periphery of the film in the aqueous solution.


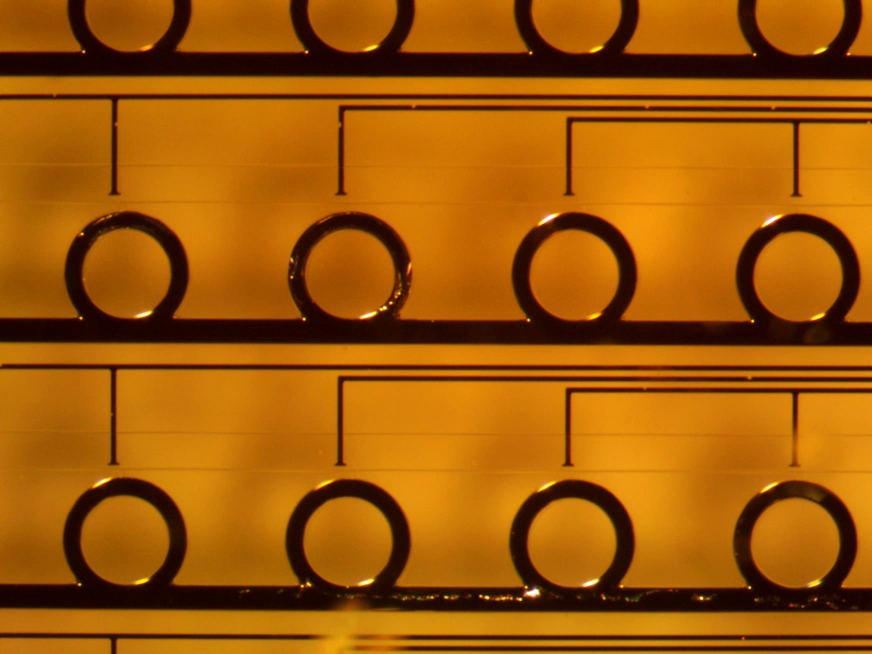


**Supplementary Video 3**. Video of the erasure and refilling process within the ring array. A potential voltage of +10 V was applied to the Au electrode immediately above a closed ring. The ground voltage was applied to an electrode well outside of the viewing window.


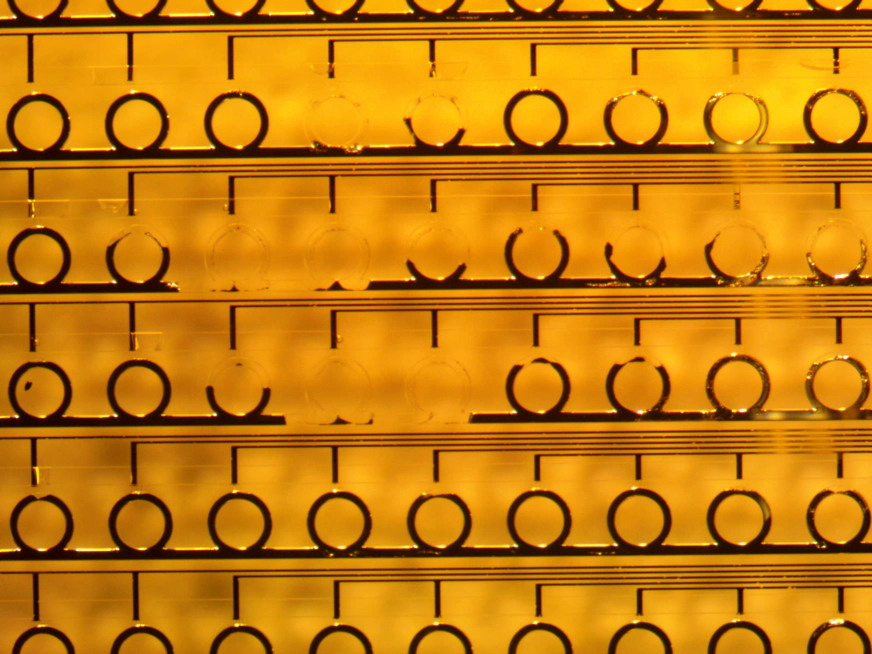


**Supplementary Video 4**. Video recording erasure of an array with random access. By controlling which electrodes have an applied voltage, the magnitude of that voltage, and the application time, we can selectively erased one ring or a group of rings each simultaneously, with the possibility of each ring being etched to a different extent. Starting with a completely filled array, a voltage of +10V was applied to the gold electrodes adjacent to the different rings for different amounts of time.
